# A rare case of atypical teratoid rhabdoid tumor (AT/RT) with homozygous SMARCB1 loss and one concurrent somatic heterozygous SMARCA4 variant

**DOI:** 10.1186/s40478-025-02129-2

**Published:** 2025-10-30

**Authors:** Ylvi Müller, Sebastian Bühner, Victoria Fincke, Katrin Mauch-Mücke, Markus J. Riemenschneider, Selma Manea, Friederike Liesche-Starnecker, Martin Hasselblatt, Sonja Dahlum, Matej Boros, Reiner Siebert, Michael C. Frühwald, Pascal Johann

**Affiliations:** 1 Pediatric and Adolescent Medicine, Faculty of Medicine, Germany Bavarian Cancer Research Center (BZKF), University of Augsburg, Stenglinstr. 2, 86156 Augsburg, Germany; 2 Department of Neuropathology, Regensburg University Hospital, Regensburg, Germany; 3 Department of Neuropathology, Medical Faculty, Institute of Pathology, University of Augsburg, Augsburg, Germany; 4 Institute of Neuropathology, University Hospital Münster, Münster, Germany; 5 Institute of Human Genetics, Ulm University and Ulm University Medical Center, Ulm, Germany; 6 The German Center for Child and Adolescent Health Ulm Site, Ulm, Germany; 7 Swabian Childrens’ Cancer Center and Bavarian Center for Cancer Research, University Childrens’ Hospital Augsburg, Augsburg, Germany

## Abstract

Atypical teratoid rhabdoid tumors (AT/RT) are characterized by a poor prognosis and a manifestation within the first 2 years of life. Genetic hallmark of these tumors is the homozygous inactivation of SMARCB1 or, in some rare cases, of SMARCA4. While heterozygous pathogenic variants of SMARCA4 have been described, inter alia, in the context of other CNS malignancies such as medulloblastoma or glioblastoma, the co-occurrence of pathogenic variants in both, SMARCB1 and SMARCA4, in the same AT/RT has to our knowledge not been reported previously. Liquid biopsy, a rapidly developing and promising technique measuring cell-free DNA (cfDNA) in body fluids such as the cerebrospinal fluid (CSF), offers a minimally invasive method to assess disease status. It has yet to be established as a standard procedure in the diagnostic workup of CNS tumors. We present the case of a three-year-old male diagnosed with an AT/RT that exhibits both biallelic alterations of SMARCB1 due to a frameshift mutation and loss of heterozygosity as well as a heterozygous missense variant in SMARCA4 presenting with early disease progression. We employed liquid biopsy successfully to monitor disease progression throughout treatment and the subsequent relapse. We highlight the ramifications that simultaneous alterations in two chromatin-modifying genes may have for tumor biology and clinical course.

## Introduction

Atypical Teratoid/Rhabdoid Tumors (AT/RT) are a highly malignant tumor entity. According to the German Childhood Cancer Registry, the relative frequency of AT/RT is approximately 0.6% of all diagnosed malignant neoplasms. AT/RT typically affects infants, with a median age at diagnosis of one year and five months. Progression of the disease is frequently driven by homozygous inactivation of SMARCB1 (INI1), and in 2–3%, of SMARCA4 [1–3]. Both genes are components of the SWI/SNF chromatin remodeling complex, which is essential for DNA damage repair, transcriptional regulation and eventually tumor suppression [4]. It is estimated that up to 35% of patients with rhabdoid tumors harbor a germline mutation [5], which is typically associated with younger age at diagnosis and inferior prognosis when compared to patients with somatic variants [6].

It is not fully understood whether SMARCA4 and SMARCB1 mutant tumors are identical in their clinical and biological characteristics and recently published evidence points at least to a differing epigenome with respect to the two mutational backgrounds: Holdhof et al. [7] identified a distinct methylation and transcriptomic phenotype in AT/RT with SMARCA4 inactivation, distinguishing them from SMARCB1 deficient cases. However, the extent to which both tumor types differ has not been comprehensively assessed—particularly not at the single cell or at the functional level. While earlier reports have suggested an even more aggressive clinical phenotype in SMARCA4 mutant cases than in SMARCB1 deficient AT/RT [7], this hypothesis so far has not been proven—owing largely to the small number of SMARCA4 deficient AT/RT, which precludes definitive conclusions.

To our knowledge, this is the first reported case of a co-occurring heterozygous SMARCA4 mutation in an AT/RT with a biallelic SMARCB1 loss. In other pediatric brain tumors, alterations in members of the SWI/SNF-complex are recurrently observed in medulloblastoma: SMARCA4 heterozygous missense variants have been identified in WNT activated and Group 3 tumors, where they cooperate with *MYC* to promote tumor formation and activate oncogenic transcription [8]. These alterations in medulloblastomas seem to be entirely somatic and it is not clear whether their mechanism of action parallels tumorigenesis in SMARCA4 -/- tumors [9].

## Case presentation

A three-year-old male presented to the pediatric emergency department with headaches and vomiting. He appeared in poor general health, with reduced nutritional status and impaired vigilance. Additionally, the patient exhibited stereotypical respiratory sounds, retching and twitching with hypersalivation. Pupils were normal in size and reactivity. The patient’s medical history, including developmental milestones, was unremarkable and there was no known family history of Rhabdoid Tumor Predisposition Syndrome (RTPS). Cranial MRI-imaging revealed a tumor in the right side of the cerebellum.

Due to the unfavorable anatomical location with proximity to the brainstem and cranial nerves, only a subtotal tumor resection with a macroscopic tumor residue was performed six days after initial presentation. A Rickham reservoir was placed for the purpose of intraventricular chemotherapy and CSF analysis of methotrexate levels as well as cytology.

Results of histopathological analysis revealed a heterogenous malignant neoplasia with embryonal characteristics and immunohistochemical loss of nuclear SMARCB1/INI1 expression, while SMARCA4/BRG1 staining was retained. Ultimately, based on methylation analyses using the Infinium MethylationEPIC BeadChip assay of Illumina, the tumor was classified as ATRT-TYR according to the Heidelberg Brain Tumor Classifier (v12.8). CNV profile in this case was without any aberrations as typical for ATRT, subgroup TYR (data not shown).

Next generation sequencing (NGS) using the TrueSight Oncology 500 assay provided by Illumina identified a variant in the SMARCB1gene: c.1175del; p.(Pro392Argfs*100), with a variant allele frequency (VAF) of 88%. Unlike typical nonsense variants that lead to early termination of protein biosynthesis, this frameshift mutation results in a prolonged protein product with an altered C-terminal.

This specific variant is not listed in databases such as HGMD professional, LOVD and gnomAD, although it has been reported once in ClinVar. According to ACMG guidelines, the variant is classified as likely pathogenic in class four with a tendency toward class five [10]. Additionally, OncoScan analysis of the tumoral DNA identified a near-complete copy number neutral loss of heterozygosity (CNN-LOH) of chromosome 22 (chr22:18,319,179–51,213,826), including the SMARCB1 gene. The CNN-LOH aligns with the high allele frequency of the SMARCB1 variant and provides an explanation for the complete loss of SMARCB1/INI1 expression.

NGS also detected a heterozygous missense variant in SMARCA4 (c.3484G > A, p.Gly1162Ser) in the tumor DNA with a VAF of 56%, affecting the C-terminal helicase domain of the protein. This variant is also not listed in the aforementioned databases. In silico prediction programs (FathmmRank, MetaLrRank, MetaRnnRank, MetaSvmRank) consistently predicted a disease-causing, deleterious effect of the alteration. However, due to insufficient data, it remains uncertain whether this SMARCA4 alteration contributes to the clinical manifestation. According to ACMG guidelines, it is categorized as a variant of uncertain significance [10]. We did not identify a second alteration of SMARCA4 in the tumor DNA by NGS or OncoScan analysis. Thus, it is unlikely that the SMARCA4 variant is causal to disease initiation but it may potentially act as a modifier of disease course. This is backed further by functional studies: The p.G1162S variant lies within the ATPase domain, which is vital for the catalytic activity of the SMARCA4 protein. Variants in this region have been shown to impair SWI/SNF complex activity and contribute to oncogenesis in multiple tumor types [11]. Table 1 provides a detailed overview of the alterations in SMARCB1 and SMARCA4 identified in the tumor tissue.

**Table 1:** Table of genetic Alterations in SMARCB1 and SMARCA4 identified in tumor tissue

SMARCB1		SMARCA4
Allele 1	Allele 2	Allele 1
SMARCB1:c.1175del;p.(P392R fs*100) (VAF: 88%), NM_003073.3	CNN-LOH on chromosome 22 (chr22:18,319,179–51,213,826)	SMARCA4; Exon 25; c.3484G > A; p.G1162S; (VAF 56%), NM_001128849.3

**Fig. 1:**
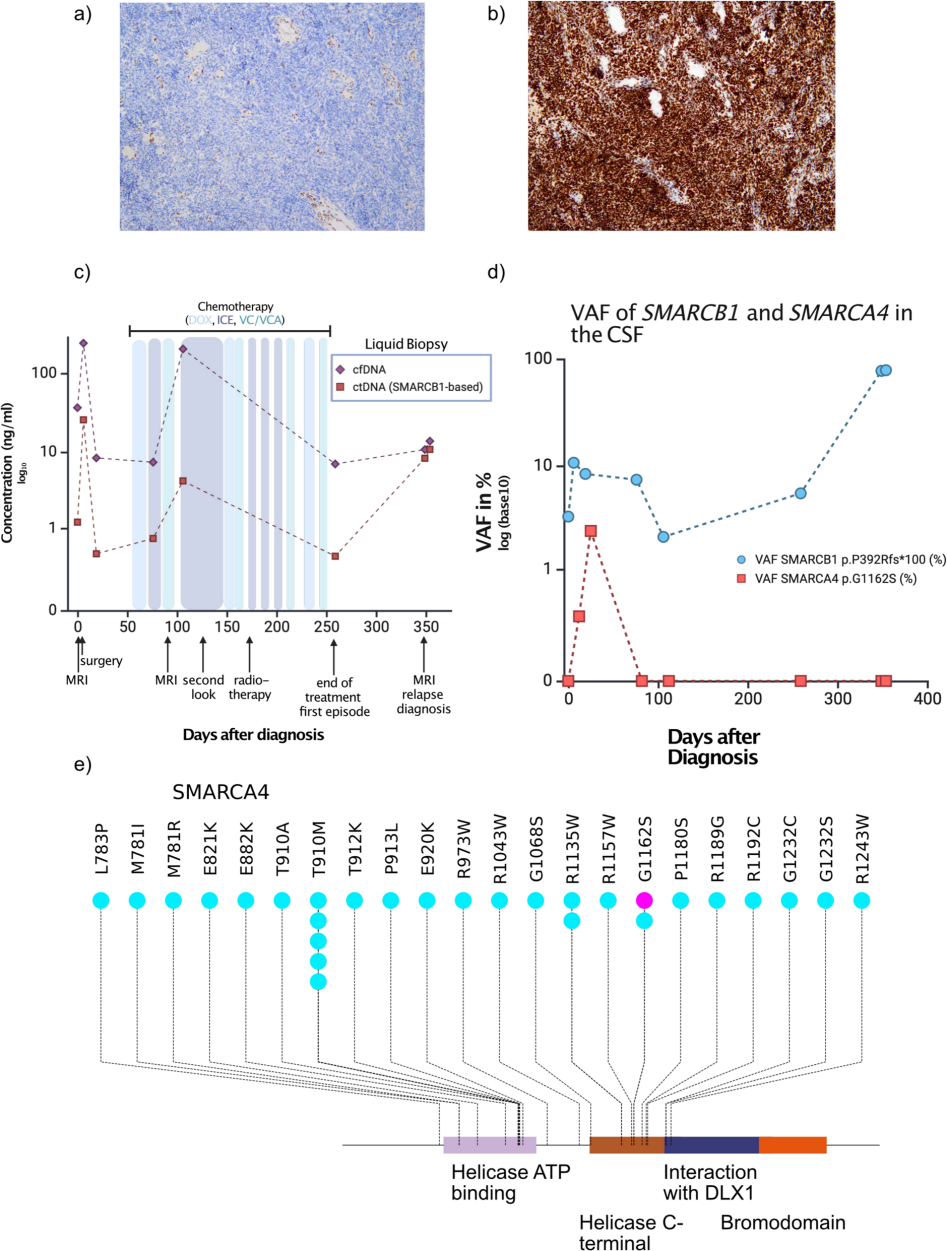
**a** Immunohistochemical staining of INI-1 in the patient case, which is lost. Panel **b** shows retained SMARCA4 staining of the same case **c** cfDNA (violet line) and ctDNA (red line) burden in the time course of the patient. **d** VAF in the course of time. **e** Lollipop plot displaying the somatic mutations of SAMRCA4 in medulloblastomas (light blue) as a reference cohort from [14] and the here described case (violet). Displayed are only the Helicase ATP binding domain, the C terminal helicase domain and the Bromodomain as the only protein domains where mutations map to.

To assess potential germline alterations, NGS and Multiplex-Ligation-dependent Probe Amplification analysis of blood-derived DNA were performed in accordance with the German Genetic Diagnostics Act. Finally, using recently developed approaches no evidence of low-level mosaicism in SMARCB1 or SMARCA4 were detected [12].These findings effectively rule out RTPS and demonstrate that the described alterations in SMARCB1 and SMARCA4 are of somatic origin (Fig. 1a).

To track disease burden based on the known SMARCB1 and SMARCA4 alterations, we included the patient in a liquid biopsy program within the frame of the BZKF (Bayerisches Zentrum für Krebsforschung) which employs isolation of cell free DNA from cerebrospinal fluid, followed by sequencing using a panel of genes, specific for pediatric neurooncology [13]. Here, we detected both the SMARCB1 frameshift variant and the SMARCA4 missense variant at a VAF varying from 2.1 to 10.7% in SMARCB1 and 0 to 2.4% in SMARCA4 depending on time (Fig. 1b).

Following surgery, the patient was enrolled in the SIOPE ATRT01 trial, receiving a total of 12 courses of chemotherapy. The chemotherapeutic treatment regimen consisted of approximately biweekly alternating administrations of doxorubicin (DOX), ifosfamide, carboplatin and etoposide (ICE) and vincristine, cyclophosphamide and actinomycin D (VCA). During the second course of VCA, actinomycin D was omitted due to the initiation of radiotherapy. Intraventricular administration of methotrexate (MTX) was planned in parallel to each course in an age-dependent fashion; however, it was ultimately administered only once via lumbar puncture during the third course of chemotherapy, first due to a cerebrospinal fistula and subsequently due to the start of radiotherapy.

To evaluate the therapeutic response, an MRI was conducted 82 days after the initial presentation following the third course of therapy. Imaging revealed remnants of the tumor around the medulla oblongata, which were initially classified as stable disease but were later reassessed as progressive disease by reference neuroradiology. Using liquid biopsy as a complementary method to MRI monitoring, we confirmed an increase in tumor burden as shown by the elevated levels of cell free DNA (cfDNA) in the CSF, but also in the ctDNA as measured by the VAF of the SMARCB1 variant (Fig. 1c, d). Given that the patient did not display an infection at this time, we assumed that this increase reflected submicroscopic tumor burden.

Based on these findings and the general recommendation to minimize residual tumor via second look surgery, a re-resection of infratentorial tumor parts was performed 3.5 months after first presentation and four courses of chemotherapy, resulting in the removal of the largest tumor mass, with only minimal residues in direct proximity to the medulla oblongata remaining in situ.

The patient subsequently underwent proton beam therapy and received the remaining chemotherapy, which was administered partially concurrently, with close disease monitoring throughout the treatment period. His treatment was completed according to protocol. After approximately nine months, at the end of therapy, an MRI scan revealed stable disease without suspicion of relapse. However, liquid biopsy monitoring at that time showed an increase of the VAF of the SMARCB1 variant (Fig. 1c) while total cfDNA concentration remained low. This increase proved to be an early indicator of disease activity.

Heterozygous missThe hitherto identified SMARCA4 variants in ATRT are exclusively homozygous and inactivating. The SMARCA4 variant described here (p.G1162S) is localized in the helicase C-terminal domain of the protein. To contextualize the variant, we reviewed the mutational data from a large-scale study on medulloblastoma [14] which identified multiple variants within the C-terminal helicase domain, including one tumor harboring the p.G1162S substitution (Fig. 1e). Of note, the same heterozygous SMARCA4 mutation has also been detected in adult T-ALL relapses (Sentis et al. Genome Biology 2020).

Three months later, follow-up imaging revealed metastatic spread, and the patient is currently undergoing treatment for early recurrence of his AT/RT. This was paralleled by an increase both of the total cfDNA but also in the VAF of the SMARCB1 gene alteration.

## Discussion

AT/RT is predominantly associated with the inactivation of SMARCB1, which is affected in nearly all cases. In rare cases, tumor development occurs due to inactivation of the SMARCA4 gene. While these tumors differ in their methylation pattern from their SMARCB1 deleted counterparts, heterozygous aberrations in SMARCA4 so far have not been implicated in the biology of rhabdoid tumors. The concurrent alteration of both SMARCB1 and SMARCA4 in a single AT/RT case represents a highly unusual molecular constellation: among 89 SMARCB1-deficient AT/RT cases analyzed at the Institute of Human Genetics, Ulm University, no co-occurring SMARCA4 variants were detected—only one patient with a heterozygous SMARCB1 and a heterozygous SMARCA4 variant was reported. Furthermore, heterozygous aberrations of SMARCA4 are findings typically observed in other pediatric brain tumors, such as medulloblastoma. Specifically, WNT activated and group 3 medulloblastoma are frequently associated with heterozygous variants in SMARCA4 [15].In addition, functional SMARCA4 or even an epigenetic regulation for increased BRG1 expression plays an essential role in tumorigenesis in other tumor entities such as group 4 and sonic hedgehog (SHH) activated medulloblastoma or glioblastoma [11]

It is difficult to foresee the progress, prognosis or treatment response of our patient, however, the early relapse after near-total resection appears rather unusual for a tumor of the ATRT-TYR subtype, particularly in a patient older than one year—a constellation generally associated with favorable outcomes [16]. Whether the detected SMARCA4 variant identified here has a relevant impact on disease course or merely acts as a bystander remains unclear.

The variant itself is listed in the Cosmic database and—as described above—displays associations with different gastrointestinal cancers (Catalogue Of Somatic Mutations In Cancer [17]), but also T-ALL and lung adenocarcinoma. The divergent VAF dynamics of SMARCA4 and SMARCB1 likely reflect their distinct biological roles: while SMARCB1 represents the truncal driver consistently maintained throughout disease evolution, the SMARCA4 variant was restricted to a subclone present at diagnosis but absent at relapse, suggesting clonal selection rather than a loss of pathogenic relevance in the primary tumor.

Notably, this patient did not harbor a germline alteration in either gene, marking the case as an example of a purely somatic mutation profile. A pivotal question raised by this case is how follow-up management can be optimized to allow for a swift response to potentially earlier or more aggressive disease progression than would be anticipated with regular tumor genetics.

Investigating the use of liquid biopsies to detect relapse earlier and in a minimally invasive manner will be valuable. The increase in the SMARCB1 mutation allele frequency that we detected at the end of therapy indeed was a herald of early relapse (Fig. 1c, d) Hence, liquid biopsy has the potential to provide additional insights for personalized treatment decisions that may contribute to improved outcomes after MRD thresholds for each entity have been established.

## Data Availability

No datasets were generated or analysed during the current study.
